# A seroepidemiological study of bovine cysticercosis in Bali and Nusa Tenggara, Indonesia

**DOI:** 10.14202/vetworld.2020.284-289

**Published:** 2020-02-15

**Authors:** Nyoman Sadra Dharmawan, I. Made Damriyasa, I. Gede Mahardika, Kadek Swastika, Luh Putu Hartiningsih, Kadek Karang Agustina

**Affiliations:** 1 Center for Study on Animal Diseases, Faculty of Veterinary Medicine, Udayana University, Denpasar, Bali, Indonesia; 2 Department of Animal Nutrition and Feed, Faculty of Animal Husbandry, Udayana University, Denpasar, Bali, Indonesia; 3 Department of Parasitology, Faculty of Medicine, Udayana University, Denpasar, Bali, Indonesia; 4 Department of Veterinary Public Health, Faculty of Veterinary Medicine, Udayana University, Denpasar, Bali, Indonesia

**Keywords:** Bali cattle, bovine cysticercosis, risk factor, *Taenia saginata*

## Abstract

**Background and Aim:**

*Taenia saginata* hazardously affects human and animal health. The distribution of this disease is found almost all over the world. The study aimed to obtain epidemiological information concerning prevalence and the distribution of bovine cysticercosis in Bali and Nusa Tenggara, Indonesia.

**Materials and Methods:**

A total of 267 community-owned Bali cattle serum samples from the provinces of Bali, West Nusa Tenggara, and East Nusa Tenggara were examined. The study was conducted by examining the serum of Bali cattle using enzyme-linked immunosorbent assay technique. Risk factors related to cysticercosis that analyzed were sex, breeding type, age, physical condition, source of drinking water, pen condition, and latrine availability.

**Results:**

Seven of 91 Bali cattle sera from all regencies/cities in Bali showed a positive result. Those positive sera were originated from Buleleng (1), Gianyar (2), Denpasar (2), and Klungkung (2). Meanwhile, four of 92 Bali cattle sera from West Nusa Tenggara and seven of 84 from East Nusa Tenggara occurred antibodies against *T. saginata*. We identified that two risk factors that influence the incidence of *T. saginata* infection in Bali cattle in Bali were the sex and the cattle breeding type.

**Conclusion:**

Through this research can be made a map of bovine cysticercosis in Bali cattle in Bali and Nusa Tenggara region. By mapping the disease, it is recommended that the animal health officers should be more accurate when conducting postmortem examination, especially on cattle from a positive region.

## Introduction

The decrease in beef production can be caused by disease factors and livestock health conditions [1]. One of the diseases that can decrease the quality of beef is an infection by the larva of *Taenia saginata* tapeworm [2]. This disease hazardously affects human health because it is zoonotic, can be transmitted from animal to human [3]. *T. saginata* metacestode generally infects cattle musculature, its disease is called cysticercosis [4]. While *T. saginata* tapeworm infects the human small intestine, its disease is called taeniasis [4]. Humans are infected by tapeworms when consuming uncooked or undercooked beef containing *Cysticercus bovis*. Conversely, the cattle will be infected by the tapeworm larvae when ingesting *T. saginata* eggs released by humans through the feces [5].

The economic impact caused by this disease is detrimental to various parties [6]. The biggest loss is suffered by the meat industry since the infected meat must be rejected and not be consumed [7]. Distribution of bovine cysticercosis is found almost all over the world, especially in rural communities, where man maintains close contact with cattle [8-10]. Human taeniasis is a parasitic infection that also has a worldwide distribution, and the highest burden was borne by communities in the developing world [11,12]. Based on research in Bali in 2002-2009, there has been found 80 cases of taeniasis from 660 people examined [13,14]. The high incidence of taeniasis in Bali is related to the habit of consuming raw beef in the form of *lawar* (a Bali traditional food contains raw blood, pork, chicken, or beef) [15].

Preventing this disease is not difficult, one of which is to break the cycle of this parasite life. However, the problem is that there are no data available on the incidence of bovine cysticercosis in cattle in Indonesia. This is due to the difficulty of diagnosing cysticercosis in the alive animals. Diagnosis of bovine cysticercosis was usually made only at postmortem examination by direct observation of the cyst through a meat inspection [16]. The cysts can sometimes be detected on the tongue of the cattle by palpation of the nodules under skin or intramuscular tissue. However, this type of detection has low sensitivity, especially in mildly infected animals [17].

Many immunodiagnostic tests have been developed for the detection of bovine cysticercosis lately. The enzyme-linked immunosorbent assay (ELISA) method was reported to give a good result [9,18]. For the test to give a good sensitivity and specificity value, this diagnostic method has been developed using the appropriate cyst antigen and is now available in commercial form. This method needs to be applied in the field to be used to do disease mapping by detecting bovine cysticercosis incidence in cattle in Indonesia, especially in the provinces of Bali and Nusa Tenggara. Bali is an area where *T. saginata* infection occurs in humans, which is always reported [14,19], while West Nusa Tenggara and East Nusa Tenggara are close to Bali. These provinces are the development areas of Bali cattle to support the needs of seeds and cattle for the whole of Indonesia [20,21].

This study aimed to collect epidemiological data such as the prevalence and distribution of bovine cysticercosis in Bali and West Nusa Tenggara, Indonesia. The specific objective of this study was to apply diagnostic techniques that have been successfully developed, then use the technique to detect cysticercosis in cattle in Indonesia, to know the exact incidence (prevalence), distribution, and risk factor of the disease.

## Materials and Methods

### Ethical approval

This research approved by the Ethical Commission for the Use of Animals in Research and Education of the Faculty of Veterinary Medicine, Udayana University, Indonesia with Ref No. 2970A/UN14.2.9/PD/2018.

### Origin of cattle and serum sampling

This study was observational in three provinces. In the province of Bali, samples were taken in each district at least 10 samples, while in West Nusa Tenggara and East Nusa Tenggara, samples were taken in animal quarantine, Bali cattle which will be sent out of the area. There is known and recorded the origin of these cows.

A total of 267 Bali cattle were sampled. In Bali area, 91 blood samples collected from eight regencies and one municipality namely Badung, Gianyar, Klungkung, Tabanan, Bangli, Karangasem, Buleleng, Jembrana, and Denpasar. For the province of West Nusa Tenggara, 92 blood samples collected from Lombok Island and Sumbawa Island. For the province of East Nusa Tenggara, 84 blood samples collected from Timor Tengah Selatan and Kupang Regencies. Serum obtained by separating the liquid fraction of whole blood by centrifugation. All samples were stored at −20°C before use.

### Detection of *T. saginata* antibodies by ELISA

Detection of antibodies against the presence of *T. saginata* infection was done using ELISA technique. The method of examination follows the official procedure issued by the Bio-X diagnostics’ cysticercosis ELISA kit antigen. *T. saginata* antigen from previous publication used as a positive control [22].

### Risk factor study

The risk factors for cysticercosis in Bali cattle in Bali studied by analyzing the relationship between cysticercosis in cattle and some data obtained from the results of questionnaires distributed to cattle owners. The owners who were the object of the study visited for interviews and data collection using a questionnaire. Data on risk factors included (1) sex; (2) breeding type; (3) age; (4) physical condition; (5) source of drinking water; (6) cage condition; and (7) latrine availability.

### Statistical analysis

The prevalence of bovine cysticercosis in Bali and West Nusa Tenggara was determined by percentage using point prevalence analysis based on ELISA results. The results are presented using the figure and given a descriptive analysis. Distribution of the disease was created based on the origin of cattle sera that detected positive *T. saginata* antibody. Risk factors analyzed by determining the odds ratio (OR).

## Results

A total of seven Bali cattle of 91 Bali originated samples contained antibodies against *T. saginata* with a prevalence of 7.69% (7/91). Four Bali cattle of 92 West Nusa Tenggara originated samples showed antibodies against *T. saginata* with a prevalence of 4.35% (7/84), while seven Bali cattle of 84 East Nusa Tenggara originated samples occurred antibodies against *T. saginata* with a prevalence of 8.33% (7/92) (Table-1).

**Table-1:** Seroprevalence of bovine cysticercosis in Bali, West Nusa Tenggara, and East Nusa Tenggara.

Bali			West Nusa Tenggara			East Nusa Tenggara		
		
Originated	±/n	%	Originated	±/n	%	Originated	±/n	%
Buleleng	1/10	10	Lembar	2/36	5.55	Timor Tengah Selatan	6/47	12.77
Karangasem	0/10	0	Gerung	1/13	7.69	Kupang	1/37	2.70
Badung	0/10	0	Jakem	0/1	0			
Tabanan	0/10	0	Labuhan Badas	0/2	0			
Gianyar	2/11	18.18	Empang	0/3	0			
Bangli	0/10	0	Plampang	1/4	25.00			
Denpasar	2/10	20	Lape	0/6	0			
Klungkung	2/10	20	Moyo Hulu	0/15	0			
			Moyo Utara	0/6	0			
			Moyo Hilir	0/6	0			
Total	7/91	7.69	Total	4/92	4.35	Total	7/84	8.33

**Figure-1:**
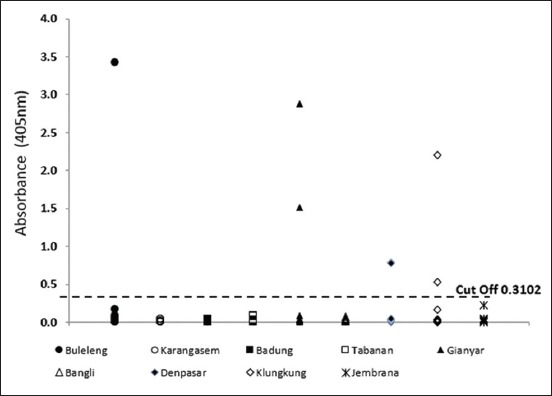
Results of enzyme-linked immunosorbent assay examination and absorbent values of antibody response against *Taenia saginata* from cattle sera obtained from Bali.

**Figure-2:**
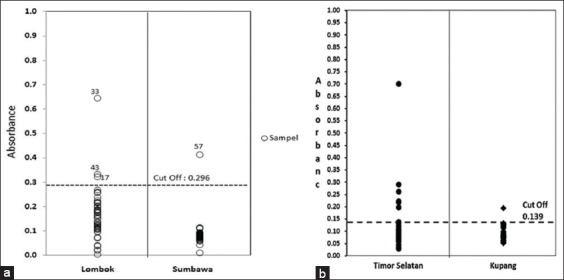
Results of enzyme-linked immunosorbent assay examination and absorbent values of antibody response against *Taenia saginata* from cattle sera obtained from West Nusa Tenggara (a) and East Nusa Tenggara (b).

Figure-1 shows the ELISA plate reader (with absorbance value 405 nm and cutoff 0.3102), where there are seven positive serum samples from Bali, which were from Buleleng (one sample), Gianyar (two samples), Klungkung (two samples), and Denpasar (two samples). Meanwhile, Figure-2a shows the results of ELISA plate reader examination of serum samples from West Nusa Tenggara (absorbance 405 nm, cutoff 0.296) and Figure-2b from East Nusa Tenggara (absorbance 405 nm, cutoff 0.139).

According to Figure-2a, there were four positive sera detected. The three positive sera are cattle sera originating from Lombok, two from Lembar Village and one from Gerung Village. Meanwhile, one other positive serum comes from Plampang Village, Sumbawa.

The location of bovine cysticercosis in Bali is in Sinabung Village of Buleleng Regency, Serangan Village of Denpasar city, Ketewel Village of Gianyar Regency, and Tangkas Village of Klungkung Regency; Lembar Village and Gerung Village in West Nusa Tenggara, Plampang Village in Sumbawa; while Kupang and Timor Tengah Selatan in East Nusa Tenggara.

### Risk factors for T. saginata infection in Bali cattle in Bali

There are several risk factors of cysticercus infection in cattle, including cattle sex, maintenance, cage structure, feed processing, clean water, and the availability of latrines for cattle owners [23,24]. In this study, we found that the risk factors for bovine cysticercosis were sex and farming type of cattle (Table-2).

**Table-2:** The odds ratio factors of cattle sex, farming type, age, physical condition, origin of drinking water, condition of the cage, and availability of latrines for cattle owners.

Risk factors	Positive		Negative		p-value	Odds ratio
	
	n	%	n	%		
Sex of cattle						
Female	1	1.54	64	98.46		
Male	6	23.07	20	76.93	0.008**	19.200
Farming type						
Breeding	2	3.13	62	96.87		
Fattening	5	18.52	22	81.48	0.025*	7.045
Age of cattle						
Young	3	7.32	38	92.68		
Old	4	8.00	46	92.00	0.903	1.101
Physical condition of cattle						
Thin and medium	5	7.46	62	92.54		
Fat	2	8.33	22	91.67	0.891	1.127
Drinking water source						
Tap water	1	2.86	34	97.14		
River	6	10.71	50	89.29	0.202	4.080
Cage floor						
Concrete	1	3.23	30	96.77		
Soil	6	10.00	54	90.00	0.275	3.333
Availability of latrines						
Available	6	7.31	76	92.69		
Not	1	11.11	8	88.89	0.687	1.583

* Significant at p *<*0.01;

** Significant at p *<*0.05

## Discussion

Bovine cysticercosis occurs most commonly in environments characterized by poor sanitation, primitive livestock husbandry practice, and inadequate meat inspection and control. According to Bedu *et al*. [25], bovine cysticercosis usually did not cause much morbidity or mortality among cattle, but it caused serious economic problems in the endemic areas due to the condemnation of meat or downgrading of carcasses.

In the current study, the prevalence of the diseases was 4.35-8.33%, which was slightly in agreement with the findings in Bahir Dar Municipal Abattoir, Ethiopia [10], in Dale Wabera District Municipal Abattoir, Western Ethiopia [26], and in Menofia Province, Egypt [27], with the prevalence of 4.2%, 6.5%, and 6.09%, respectively. However, it was slightly greater than the findings around Asella Town, Tiyo Woreda, South East Ethiopia and at Zeway Municipal Abattoir, Ethiopia, with the prevalence of 1.2% and 3%, respectively [25,28], and conversely lower than findings of Hirpha *et al*. [29] in and around Halaba Kulito Town, South Ethiopia, with the prevalence of 8.6%.

According to Laranjo-González *et al*. [24], reports on prevalence bovine cysticercosis in European countries were mostly from Western and Central Europe. The prevalence which has been calculated based on meat inspection was generally low (below 6.2%) and varied between and within countries. It further stated that with serology and detailed meat inspection provided a higher prevalence range (0.41-14%). The different prevalence reported in these studies might be due to several factors, of which husbandry systems, hygiene differences, and eating habits were among the most important [26]. In addition, the diagnosis of bovine cysticercosis by meat inspection underestimates the true prevalence, especially when the infection is light [26,30].

The results of this study were also corresponding and reciprocal with the results of studies on the prevalence of taeniasis in humans in Bali. The taeniasis and cysticercosis research had been conducted since 2002 which was a cooperation between Udayana University, Bali, with Asahikawa Medical University, Japan, and the Ministry of Health Republic of Indonesia. It showed that most of *Taenia* tapeworm identified by the epidemiological study was *T. saginata*. Most of these infections occur in Gianyar Regency [14,19]. On the other hand, in the report of the Directorate General of Animal Health Ministry of Agriculture, it is known that in 1977, the prevalence of cysticercosis in cattle in four regencies in Bali, namely, Badung, Gianyar, Klungkung, and Tabanan was 3.3%, 16.9%, 1.2%, and 8.3%, respectively [31].

Our study leads to a further mapping of bovine cysticercosis in Bali and West Nusa Tenggara. Based on the mapping, we can conceptualize cysticercosis prevention strategies in the region. One of the concepts that must be applied is to further improve the method of postmortem examination of cattle before slaughter to provide healthy meat for public consumption. In accordance with the results of workshops that have been done, in addition to the active and passive taeniasis and cysticercosis surveillance, activities such as health surveillance of *lawar* sellers and their family members need to be done periodically. Furthermore, there is a need to improve public health education, especially to children school with a focus on personal hygiene, environmental sanitation, and good cattle raising [14].

Table-2 shows that the factor that has the highest risk of cysticercosis in Bali cattle in Bali was sex. Male cattle have a risk of 19.2 times greater cysticercosis infection than female cattle (p<0.01). The results of this study correspond to the research reported by Edao *et al*. [28] who conducted a study on the prevalence of *T. saginata* infection in cattle and taeniasis status in humans around Asella city, Southeast Ethiopia. From the results of his research, it is known that sex was one of the risk factors for cysticercosis in cattle. Risk factors associated with the high incidence of cysticercosis in Ethiopian cattle were local cattle, adult age, and male sexes [28]. Hirpha *et al*. [29] also reported that there was a significant relationship between sex and the prevalence of *T. saginata* infection in cattle.

However, the results of this study differ from those of Bedu *et al*. [25] which stated that there was no significant difference between the prevalence of cysticercosis in cattle with risk factors for sex and animal origin. The same thing also reported by other researchers [32]. Ydnekew *et al*. [33] who observed 312 cattle at Mekelle animal slaughterhouse in Ethiopia found 21 cattle infected with *T. saginata*, but the sex did not significantly influence (p>0.05) on the incidence of infection. These differences were in addition caused by differences in environment/agro-climate, also due to differences in sampling locations [28,34].

The results of this study also found that there was a significant difference (p<0.05) in risk factors related to cattle farming type (Table-2). Fattening was a risk factor that is 7.1 times more risky to get an infection than breeding. The fattening cattle samples used in this study generally remained in the cage. The cattle receive food and drinks brought by the owners. Thus, there was no other choice for that cattle to consume feed given by the owner. This condition causes fattening cattle to have a greater chance of being infected with *T. saginata* if the feed or drinking water given is contaminated by *T. saginata* eggs. Laranjo-González *et al*. [24] reported that one of the potential risk factors associated with cysticercosis in cattle was the access of livestock to drinking water or animal feed contaminated with *T. saginata* eggs.

Based on the results of interviews conducted in this study, we found that six out of seven cows (85.71%) were positively infected with *T. saginata* that obtained drinking water sourced from the river. OR analysis shows that drinking water originating from the river has a risk of 4 times greater cysticercosis infection than water originating from tap water. Rivers in Bali, especially in rural areas, are still used for bathing, washing, and latrines.

The availability of clean water around the farm not only affects the cleanliness of the cage but also the security for consumption. It shows that water availability is one of the important factors in housing sanitation systems and availability for drinking water. EFSA [23] suggests that the unavailability of clean water greatly affects cysticercosis in livestock. Boone *et al*. [35] found that the unavailability of clean water was one of the risk factors for cysticercosis in cattle. The same problem was also found in livestock in developed countries. The results of research conducted in Belgium, Italy, and Spain, also mentioned that one of the risk factors for infection with *T. saginata* in cattle was a source of drinking water. It was reported that the source of the spread came from workers who came from developing countries as carriers of *T. saginata*. Workers used pool facilities and pollute the water with *T saginata* eggs or proglottid when they swim. The pool water and also water from the septic tank drainage were then used to water vegetables or grass for animal feed [24].

## Conclusion

The prevalence of bovine cysticercosis in Bali cattle in Bali was 7.69%, in West Nusa Tenggara was 4.35%, and in East Nusa Tenggara was 8.33. The distribution of this disease in Bali included Sinabung Village of Buleleng Regency, Ketewel Village of Gianyar Regency, Serangan Village of Denpasar city, and Tangkas Village of Klungkung Regency; in West Nusa Tenggara included Lembar Village and Gerung Village in West Lombok, and Plampang Village in Sumbawa; and in East Nusa Tenggara included Timor Tengah Selatan and Kupang. The risk factors that influence the incidence of *T. saginata* infection in Bali cattle in Bali were the sex and the cattle breeding type.

## Authors’ Contributions

NSD: Designed the research, sample collection, laboratory work, analyze the data, and writing the manuscript. IMD: Designed the research and analyzed the data. IGM: Designed the research and analyze the data. KS: Sample collection and laboratory work. LPH: Sample collection, laboratory work, and analyze the data. KKA: Analyze the data and writing the manuscript. All authors read and approved the final manuscript.
